# Commentary: Plant Auxin Biosynthesis Did Not Originate in Charophytes

**DOI:** 10.3389/fpls.2016.00158

**Published:** 2016-02-16

**Authors:** Chunyang Wang, Si-Shen Li, Guan-Zhu Han

**Affiliations:** ^1^State Key Laboratory of Crop Biology, Shandong Agricultural UniversityTai'an, China; ^2^Jiangsu Key Laboratory for Microbes and Functional Genomics, Jiangsu Engineering and Technology Research Center for Microbiology, College of Life Sciences, Nanjing Normal UniversityNanjing, China

**Keywords:** auxin biosynthesis, phylogenetics, charophytes, horizontal gene transfer, comparative genomics

The TRYPTOPHAN AMINOTRANSFERASE OF ARABIDOPSIS (TAA) family of aminotransferases and the YUCCA (YUC) family of flavin monooxygenases are required for the biosynthesis of auxin (Mashiguchi et al., 2011). However, the origin of TAA-YUC auxin biosynthesis pathway is under hot debate recently (Wang et al., 2014; Yue et al., 2014; Turnaev et al., 2015). By similarity searches and phylogenetic analyses, Yue et al. did not find TAA and YUC homologs in any algal group (Yue et al., 2014). On the contrary, we found TAA and YUC protein homologs in the genome of *Klebsormidium flaccidum*, a charophyte alga, and proposed that plant auxin biosynthesis might originate in charophytes (Wang et al., 2014). More recently, Turnaev et al. (2015) reanalyzed the structures and phylogenetic relationship of TAA family proteins and claimed *K. flaccidum* TAA-like protein (kfl00051_0080) is more closely related to alliinases than tryptophan aminotransferases. However, we believe this represents a common misinterpretation of phylogenetic tree, which leads to erroneous inferences of ancestry and evolutionary relationship.

First, Turnaev et al. missed several TAA protein homologs in Choanoflagellida. We employed the BLASTP algorithm with *Arabidopsis thaliana* TAA1 protein as the query and found significant hits in *Salpingoeca rosetta* [XP_004988548, *e* = 10^−37^, identity = 97/317 (31%)] and *Monosiga brevicollis* [XP_001746485, *e* = 10^−34^, identity = 86/290 (30%)]. Also, Turnaev et al. missed many TAA protein homologs in bacteria [e.g., WP_012083818 of *Sulfurovum* sp., *e* = 2 × 10^−8^, identitiy = 75/302 (25%)] and archaea [e.g., WP_006652091 of *Natrialba hulunbeirensis*, *e* = 9 × 10^−7^, identity = 82/323 (25%)]. When the identity of protein pairs is higher than 25%, and the number of residues aligned is higher than 150, evolutionary relatedness could be convincingly inferred (Chung and Subbiah, 1996; Rost, 1999). It appears that these additional eukaryotic and prokaryotic proteins are homologous to *A. thaliana* TAA1 protein. Missing bacteria and archaea homologs leads to the absence of outgroup taxa to root the TAA phylogenetic tree (Figure 1A). Also, one key node (the clade of land plant alliinases, kfl00051_0080, and the *Capsaspora owczarzaki* sequence) is weakly supported (posterior probability: 0.463), making the deep relationship of plant and non-plant eukaryote TAA proteins unresolved. However, our phylogenetic tree with more non-plant eukaryote and prokaryote TAA-like proteins is more strongly supported in terms of posterior probability (see Figure 1D in this paper and Figure 1A in Wang et al., 2014).

**Figure 1:**
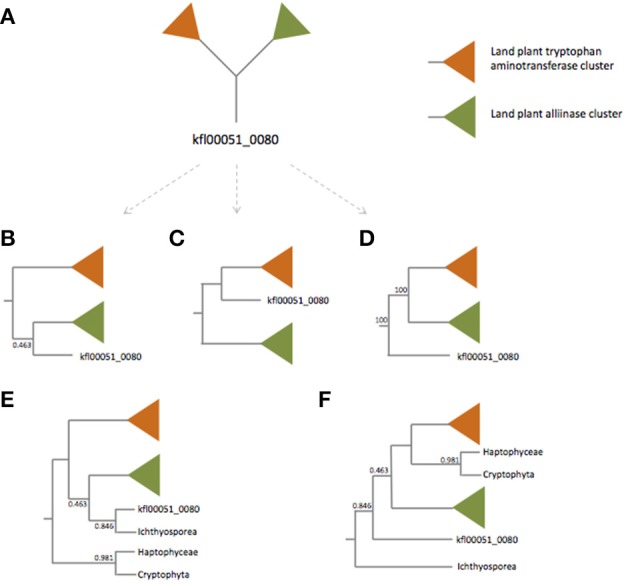
**Simplified unrooted (A) and rooted tree (B–D) of TAA family proteins**. For the unrooted tree **(A)**, there are at least three rooted trees **(B–D)**. The orange and green triangles represent land plant tryptophan aminotransferase and alliinase clades respectively. Trees **(E,F)** are rooted trees with the Haptophyceae and Cryptophyta sequences and the Ichthyosporea sequence as the outgroups, respectively. Trees **(B,E)** are modified from Turnaev et al. (2015). Tree **(D)** is modified from Wang et al. (2014).

Most importantly, what Turnaev et al. present is an unrooted phylogenetic tree. Based on the unrooted phylogenetic tree, they claimed the kfl00051_0080 from *K. flaccidum* belongs to the land plant alliinase clade. Unfortunately, this claim results from a misinterpretation of phylogenetic tree, i.e., confusion between unrooted tree and rooted tree. For an unrooted tree, the common ancestor to all the taxa is not identified. The root can be placed along any of the branches of an unrooted tree. For an unrooted tree, there are 2*T*-3 possible rooted trees, where *T* represents the number of taxa within the tree. To be simple, let's ignore the branches within land plants and the nodes of non-plant eukaryotes (the key node is not well supported; Figure 1A). Without a prior knowing of where the root is, the simplified unrooted phylogenetic tree of TAA proteins can be “transformed” into three rooted trees (Figures 1B–D); it should be noted that only one of them is correct. Turnaev et al. arbitrarily took an unrooted tree as a rooted tree and concluded that *K. flaccidum* TAA protein is more closely related to plant alliinases (Figure 1B). Even if we assume Turnaev et al.'s tree is a rooted tree with Cryptophyta and Haptophyceae as the outgroups (Figure 1E). Then the problem is why they used Cryptophyta and Haptophyceae sequences as the outgroups, but not Ichthyosporea sequence (Figure 1F). If we use the Ichthyosporea sequence as the outgroup, the *K. flaccidum* kfl00051_0080 is basal to land plant alliinases and tryptophan aminotransferases (Figure 1F). Nevertheless, our phylogenetic analysis with bacterial and archaeal sequences reveals where the root is (see Figure 1D in this paper and Figure 1A in Wang et al., 2014). It appears that the *K. flaccidum* kfl00051_0080 is equally related to land plant alliinases and tryptophan aminotransferases.

Based on the phylogenetic analysis, Turnaev et al. (2015) conclude “land plants and the ancestor of *K. flaccidum* acquired *TAA*-like and alliinase genes independently through a horizontal gene transfer from non-plant taxa.” Again this claim is based on an unrooted tree. The directionality of their phylogenetic tree is unknown and thus the claim is not verified. Even we assume that Turnaev et al.'s tree is rooted as discussed above, there are not two HGT events from non-plant taxa, because the most parsimonious explanation of Ichthyosporea sequence should be HGT from plant to Ichthyosporea. Our phylogenetic analysis with bacteria and archaea clearly shows that all the alliinase/aminotransferase genes originated from a single horizontal gene transfer event (Wang et al., 2014). Our explanation (one gain) is more parsimonious than Turnaev et al.'s (scenario 1: two independent HGT events; scenario 2: one gain, one duplication, one loss, etc.).

The two clades of land plant TAA family proteins arose from an ancient gene duplication event that occurred after the divergence of *K. flaccidum* from plants but before/around the emergence of land plants. In terms of functionality origin and evolution, there are at least two possible trajectories, subfunctionalization (the proto-gene represented by kfl0051_0080 has both functions) and neofunctionalization (either alliinase or aminotransferase function is newcomer). However, these hypotheses are based on the assumption that the so-called alliinases do not participate in the biosynthesis of auxin, which we still do not know. It is likely that both clades of TAA family proteins play a role in the auxin biosynthesis, since the core residues of substrate binding are conserved among tryptophan aminotransferases and alliinases of land plants and *K. flaccidum* (Figure S1; He et al., 2011). We agree that further experimental work could resolve the controversy.

Nevertheless, this case might point to a general problem when researchers from outside the field of evolutionary biology come into phylogenetic analysis. Nowadays phylogenetic analysis has become more and more frequently used in modern biology, including plant science. How to interpret phylogenetic tree is becoming a challenge (Baum et al., 2005). It should be noted that nearly all the tree inference (Neighbor Joining, Maximum Likelihood, Bayesian, etc.) methods produce unrooted trees (Philippe et al., 2011). Often the tree displayed by software is intuitively taken as rooted tree, which is likely to lead to misinterpretation of phylogenetic tree and errors, especially the common fallacy that taxa are assigned to the wrong clade.

## Author contributions

GH, CW, and SL conceived the study. GH wrote the manuscript.

### Conflict of interest statement

The authors declare that the research was conducted in the absence of any commercial or financial relationships that could be construed as a potential conflict of interest.
