# Supplementary material for: Commentary: Plant Auxin Biosynthesis Did Not Originate in Charophytes
Source: Front Plant Sci. 2016 Feb 16;7:158. doi: 10.3389/fpls.2016.00158 (PMC4754409; doi:10.3389/fpls.2016.00158)
Supplement: Supplementary file 1 [file Image1.PDF]

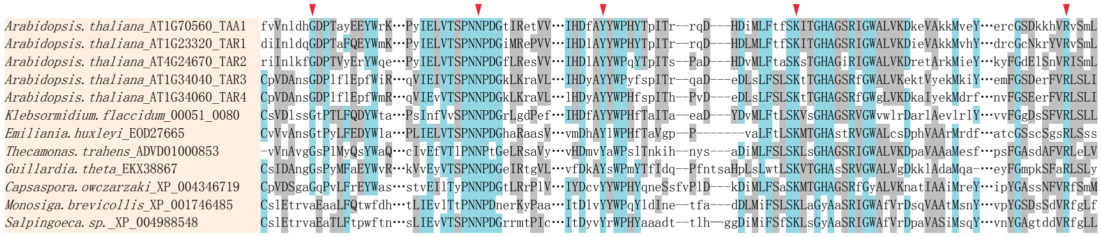

Fig. S1. The alignment of representative TAA family of proteins. Red triangles represents the core residues of substrate binding.
